# Collision tumor at the rectum consisting of a neuroendocrine carcinoma and adenocarcinoma: A case report

**DOI:** 10.1016/j.amsu.2022.103748

**Published:** 2022-05-10

**Authors:** Jaspreet Nannar, Alyssa Anciro, Atul K. Nanda

**Affiliations:** a Medical Student, St. George's University School of Medicine, Humboldt Park Health, 1044 N Francisco Avenue, Chicago, IL, 60622, USA; b Chairman of Surgery, Humboldt Park Health, 1044 N Francisco Avenue, Chicago, IL, 60622, USA

**Keywords:** Collision tumor, General surgery, Oncology, Case report

## Abstract

**Introduction and Importance:**

Neuroendocrine carcinoma of the colon and rectum account for less than 1% of all colorectal malignancies. Here we report a case of a collision tumor at the rectum consisting of a neuroendocrine carcinoma and adenocarcinoma.

**Case presentation:**

A 46-year-old asymptomatic female with severe anemia was referred by her primary care physician for admission. Initial imaging showed several innumerable heterogenous hypodense lesions within the liver. Following a colonoscopy and the excision of a rectal mass, the histopathology report revealed a Collision tumor.

**Clinical discussion:**

There are no specific clinical features or imaging findings. The diagnosis is made post operatively dependent on histopathology.

**Conclusion:**

It is essential to report these cases to advance a further understanding of the behaviour of these tumors, in addition to develop evidence-based guidelines and treatment strategies.

## Highlights

• Collision tumors are rare consisting of 2 independent tumors co-existing.
• Diagnosis is post-operatively and dependent on histology.
• No current guidelines for treatment. Management focuses on the predominant tumor.

## 1 Introduction

The most common malignancy of the rectum is adenocarcinoma. Neuroendocrine carcinoma (NEC) of the colon and rectum accounts for <1% of all colorectal malignancies [1]. NEC are aggressive and tend to metastasize [1].

Here we present a case of a collision tumor consisting of a neuroendocrine carcinoma and adenocarcinoma at the rectum. Collision tumor is two or more distinct neoplasms that develop adjacent to one another [2]. Whereas composite tumors originate from the same neoplastic clonal proliferation [1]. However, in literature collision and composite tumors have been used interchangeably due to the lack of satisfactory explanations of their origin [3]. Histologically, collision tumors maintain a distinctive border with no or minimal intermixing between the different types of tumors, whereas composite tumors intermix [[1], [3]].

This case report has been reported in line with the SCARE Criteria [4].

### 1.1 Case description

A 46-year-old female with hemoglobin of 6.6 at her primary care physician's office was referred for admission at a community hospital. Past medical history includes hypertension, mental disability, uterine fibroids, hypothyroidism, small bowel obstruction, anemia and constipation. Patient's family history was significant for cancer, however the type was undetermined. On physical examination, the patient's abdomen was distended with tympany in the upper and central abdomen, tenderness in the lower abdomen, in addition to an enlarged and irregular uterus. Patient did not allow for a digital rectal examination. On blood test, there was leukocytosis, anemia, thrombocytopenia. The levels of the tumor markers were elevated as follows: carcinoembryonic antigen, 21.9, CA 19–9, 66, CA 125, 39.

**Image 1:**
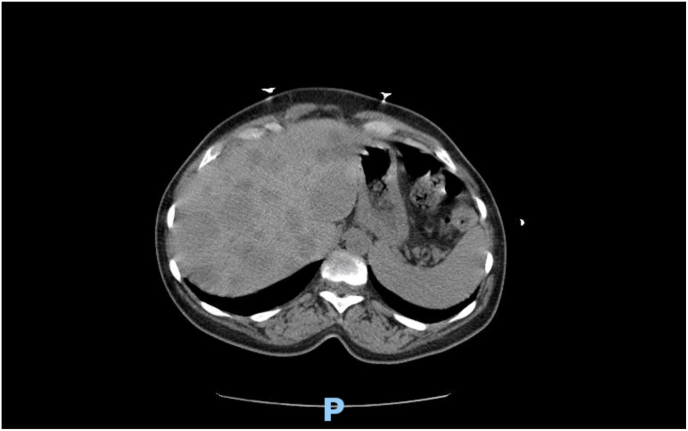
Axial plane of CT scan of abdomen.

**Image 2:**
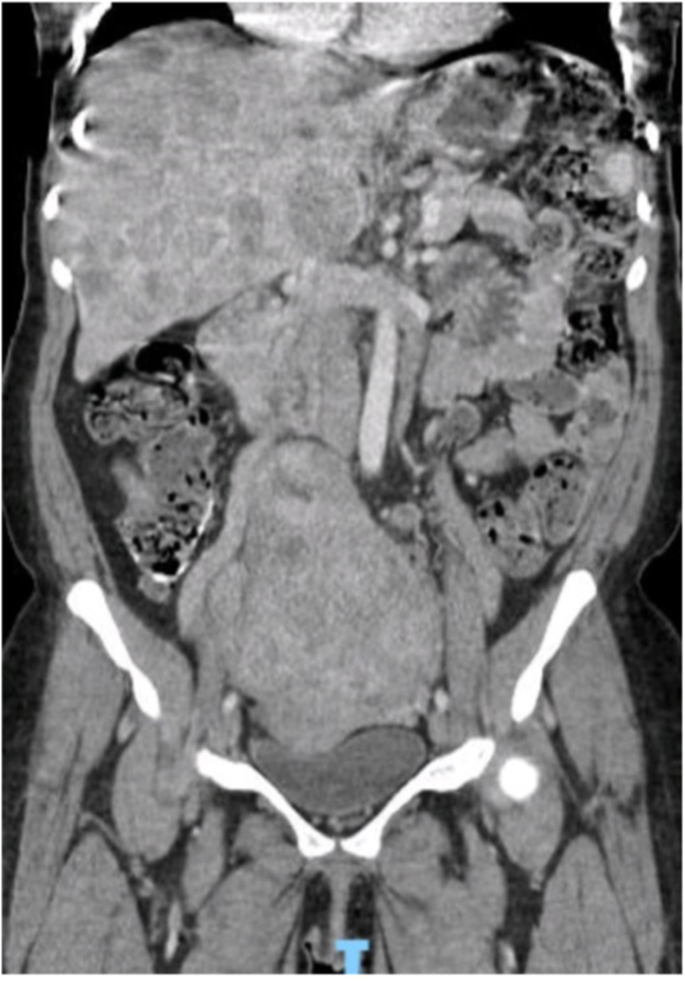
Coronal plane of CT scan of abdomen.

A computed tomography scan of the abdomen showed innumerable heterogenous hypodense lesions within the liver and mildly enlarged periportal and pelvic lymphadenopathy concerning for metastatic nodal disease (Image 1, Image 2). She subsequently underwent a colonoscopy for anemia that showed a malignant appearing, mass approximately 6 cm in size, close to the anal verge. The biopsy revealed a poorly differentiated neuroendocrine carcinoma associated with a villous adenoma.

**Fig. 1:**
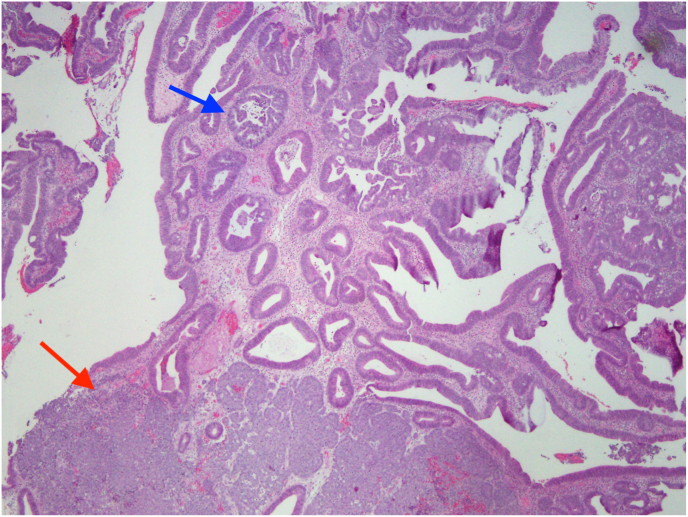
Low power view showing the adenocarcinoma (blue arrow) and neuroendocrine carcinoma (red arrow). (For interpretation of the references to color in this figure legend, the reader is referred to the Web version of this article.)

Immunohistochemically the tumor was positive for Cytokeratin AE1/AE3 and p16, synaptophysin 4+ and Ki67 85%. The size of the villous adenoma prevented complete excision during the colonoscopy. Due to the risk of progression of adenoma to a rectal adenocarcinoma, the patient was planned for a transanal excision by an experienced general surgeon. Examination under anesthesia with proctoscopy revealed a sessile, friable, wide based anorectal mass measuring 4.5 cm × 3 cm wide. The mass was infiltrating deep to the submucosa and located in the right anal canal. The histopathology report revealed a high-grade neuroendocrine carcinoma with glandular differentiation. The staining pattern supports a Collision tumor with a malignant neuroendocrine and features of a collision tumor with a smaller adenocarcinoma (Fig. 1). Immunohistochemically positive for synaptophysin, CD56, and CDX2 in the glandular area but negative in the solid tumor and Ki67 95%.

The patient also underwent an ultrasound guided biopsy of liver lesions. The pathology report revealed the masses were metastatic poorly differentiated carcinoma which were consistent with the history of neuroendocrine collision tumor from the rectum. Patient was subsequently readmitted to the ICU for altered mental status and sepsis. Her admission course was unremitting, and she unfortunately passed away prior to receiving chemotherapy consisting of Etoposide and Carboplatin.

## 2 Discussion

Collision tumors are two co-existing but independent tumors [5]. In contrast, composite tumors originate from the same neoplastic clonal proliferation [1]. However, often in literature, collision and composite tumors are used interchangeably because currently there are no satisfactory explanations about the origin [3]. The theories that have been proposed include (1) accidental meeting of two co-existing neoplasms that developed independently, (2) a single carcinogen leads to the development of two colliding neoplasms, and (3) stimulation of tumor-to-tumor carcinogenesis – one tumor inducing the development of a second primary tumor [6]. These theories assume that collision tumors develop from two different neoplasms. However, experimental data contradicts this and indicates some collision tumors develop from a single cell and then during tumorigenesis differentiates into two histological types [ [6,7]]. *Milne* et al. performed a p53 and loss of heterozygosity analysis in cell components of collision and composite tumors which showed they shared the same mutations [7]. Similarly, *Fukui* et al. also reported similar results while studying a neuroendocrine-gastric adenocarcinoma collision tumor [8]. Clinically, data show that lymph node metastasis shows a collision pattern. Research theories, however, suggest that only one element of the collision tumor will metastasize, therefore indicating the metastasis occurred before differentiation [9,10].

The clinical presentation of collision tumors is non-specific and varies depending on the location. Rectal neuroendocrine neoplasms tend to be asymptomatic, but patients can present with rectal bleeding, change in bowel habits and pain [11]. There are no specific radiological findings [2]. The diagnosis is difficult to make pre-operatively and is only confirmed post-operatively and depends on histology [2]. Spagnolo and Heenan proposed the following diagnostic criteria for collision tumors: (1) clear identification of two different tissue types (2) separation of the 2 components, (3) at the point of mixing, some transitional patterns may be seen [12]. Immunohistochemical study is also beneficial, especially when differentiating between a collision and composite tumor [13]. Histologically, collision tumors will show a sharp delineation between the tumor types, whereas composite tumors will not and there will be intermixing of the tumors [2]. There is poor evidence based clinical management and treatment strategies available due to the infrequency of collision tumors. As a result, currently there are no definitive guidelines available for treatment. Generally, treatment is primarily focused on the tumor type that is more histologically aggressive or has predominant behavioural features [2]. For neuroendocrine carcinomas, the first line therapy includes Irinotecan and Cisplatin, which is followed by second line therapy with etoposide and carboplatin [1]. Colorectal neuroendocrine carcinoma has a poor prognosis due to the aggressiveness and late detection [1]. At 5 years, the median survival rate and relative survival (%) is 7.1–14.7 months and 8–16% respectively. In contrast to colorectal adenocarcinoma the median survival rate and relative survival (%) is 36 months and 50.2% respectively [1].

According to the World Health Organization, neuroendocrine neoplasms are divided into neuroendocrine tumors and neuroendocrine carcinomas [14]. Neuroendocrine tumors are well differentiated and can be further separated based on their grade (G1, G2, G3). In contrast, neuroendocrine carcinomas are not graded, and are all classified as high grade but can further be divided into small cell or large cell types [14].

## 3 Conclusion

Collision tumors of the rectum are rare. Further reporting of such cases is essential to develop a deeper understanding of the behaviour, treatment options and overall prognosis.

## Ethical approval

N/a.

## Sources of funding

None.

## Author contribution

Jaspreet Nannar - Writing – Original Draft, Writing – Review & Editing. Alyssa Anciro- Writing – Review & Editing. Atul K Nanda - Methodology, Writing – Review & Editing, Supervision.

## Research registration

N/a.

## Guarantor

Jaspreet Nannar, Alyssa Anciro, Atul K Nanda.

## Consent

Obtained from patient's surrogate decision maker – was not available in person and that's why consent over the phone was taken.

## Informed consent

Written informed consent was obtained from the patient for publication of this case report and accompanying images. A copy of the written consent is available for review by the Editor-in-Chief of this journal on request.

## Provenance and peer review

Not commissioned, externally peer-reviewed.

## Declaration of competing interest

None.

## References

[1] Yoshida T., Kamimura K., Hosaka K., Doumori K., Oka H., Sato A., Terai S. (2019). Colorectal neuroendocrine carcinoma: a case report and review of the literature. World J. Clin. Case.

[2] Schizas D., Katsaros I., Michalinos A., Damaskos C., Garmpis N., Ntomi V., Tsaroucha A.K. (2018). Collision tumors of the gastrointestinal tract: a systematic review of the literature. Anticancer Res..

[3] Meşină C., Vasile I., Ciobanu D., Calotă F., Gruia C.L., Streba L., Tarniţă D.N. (2014). Collision tumor of recto-sigmoidian junction–case presentation. Rom. J. Morphol. Embryol..

[4] Agha R.A., Franchi T., Sohrabi C., Mathew G., Kerwan A., Thoma A., Mei Z. (2020). The SCARE 2020 guideline: updating consensus surgical CAse REport (SCARE) guidelines. Int. J. Surg..

[5] Roh Y.H., Lee H.W., Kim M.C., Lee K.W., Roh M.S. (2006). Collision tumor of the rectum: a case report of metastatic gastric adenocarcinoma plus primary rectal adenocarcinoma. World J. Gastroenterol.: WJG.

[6] Michalinos A., Constantinidou A., Kontos M. (2015). Gastric collision tumors: an insight into their origin and clinical significance. Gastroenterol. Res. Pract..

[7] Milne A.N., Carvalho R., Van Rees B.P., Van Lanschot J.J., Offerhaus G.J.A., Weterman M.A. (2004). Do collision tumors of the gastroesophageal junction exist?: a molecular analysis. Am. J. Surg. Pathol..

[8] Fukui H., Takada M., Chiba T., Kashiwagi R., Sakane M., Tabata F., Fujimori T. (2001). Concurrent occurrence of gastric adenocarcinoma and duodenal neuroendocrine cell carcinoma: a composite tumour or collision tumours?. Gut.

[9] Jernstrom P., Murray G.C. (1966). Synchronous double primary lymphosarcoma and adenosarcoma (collision tumor) of the stomach with cancer‐to‐cancer metastasis. Cancer.

[10] Parks T.G. (1970). Malignant carcinoid and adenocarcinoma of the stomach. J. Br. Surg..

[11] Bertani E., Ravizza D., Milione M., Massironi S., Grana C.M., Zerini D., Fazio N. (2018). Neuroendocrine neoplasms of rectum: a management update. Cancer Treat Rev..

[12] Spagnolo D.V., Heenan P.J. (1980). Collision carcinoma at the esophagogastric junction: report of two cases. Cancer.

[13] Miyamoto R., Kikuchi K., Uchida A., Ozawa M., Kemmochi A., Sano N., Yamamoto M. (2018).

[14] Nagtegaal I.D., Odze R.D., Klimstra D., Paradis V., Rugge M., Schirmacher P., Washington K.M., Carneiro F., Cree I.A. (2020). The 2019 WHO classification of tumours of the digestive system. Histopathology.

